# Induction of cachexia in mice

**DOI:** 10.1038/sj.bjc.6690258

**Published:** 1999-03

**Authors:** M Tisdale


*British Journal of Cancer* (1999) **79**(9/10), 1620–1628
© 1999 Cancer Research Campaign
Article no. bjoc.1998.0258

Letters to the Editor

### Induction of cachexia in mice

**Sir,**

We have read with great interest the article by Cariuk et al (*Br J Cancer* 1997 **76**: 606–613) entitled 'Induction of cachexia in mice by a product isolated from the urine of cachectic cancer patients'. It is interesting to read about so-called cachexia-inducing factor(s). The results obtained by Cariuk et al (1997) in their exhaustive and well-designed study reveal that there is a substance responsible for cachexia specifically occurring in cancer patients.

As reported in their prepublished papers, they described a proteoglycan of $M_r$ 24 000, detected by Western blotting using serum from mice bearing a cachexia-inducing tumour (MAC16), which was present in the urine of patients with cancer cachexia but absent from the urine of normal subjects (McDevitt et al, 1995; Todorov et al, 1996). Significantly, this detected proteoglycan is most likely a cachexia-inducing factor, according to the results obtained from their studies in mice.

The authors have looked for the proteoglycan only in the urine of the cachectic cancer patients, but not in the serum. They have not taken the potential serum levels into account, which would be more valuable. It is possible for such a proteoglycan to have undergone renal metabolism or been affected by glomerular-filtration rate, before it reaches the urine. It is interesting, and somewhat questionable, to read that the proteoglycan capable of inducing cachexia in mice and the one suspected of being responsible for cachexia induction in humans are of the same molecular weight. Moreover, Cariuk et al have isolated the former from the serum but the latter from the urine. It is well known that the kidney plays an important role in stabilization of the concentration and turnover of oligopeptides and proteins in the extracellular fluid matrix, and that it acts essentially as a passive barrier against loss of intermediate and high molecular weight circulating proteins (Johnson and Maack, 1989). This raises the question whether there is a protein of higher molecular weight accompanying the proteoglycan of $M_r$ 24000, described in the urine of cachectic cancer patients, that has more significance in the aetiology of cachexia in cancer patients and that is eliminated during its travel through the kidney. Looking at the serum of such cachectic cancer patients (instead of their urine) for isolation of proteins suspected of being cachexia-inducing factors would not be difficult, but would help to clarify this obscurity and would increase the value of the results obtained by Cariuk et al (1997).

In addition, we would like to implicate the importance of monoclonal antibodies, produced against the factors that induce cachexia, in the prevention of cachexia. Although they do not have any positive effects on the treatment of the underlying cancer they can be helpful in reducing the negative metabolic effects of cachexia to the body.

*İ Güllü and S Marangoz, Hacettepe University, Institute of Oncology, 06100 Sihhiye, Ankara, Turkey*

**REFERENCES**

Cariuk P, Lorite MJ, Todorov PT, Field WN, Wigmore SJ and Tisdale MJ (1997) Induction of cachexia in mice by a product isolated from the urine of cachectic cancer patients. *Br J Cancer* **76**: 606–613
Johnson V and Maack T (1989) Renal tubular handling of proteins and peptides. In *Textbook of Nephrology*, Massry SG and Glassock RJ (eds), pp. 97–102. Williams and Wilkins: Baltimore
McDevitt TM, Todorov PT, Beck SA, Khan SH and Tisdale MJ (1995) Purification and characterization of a lipid-mobilizing factor associated with cachexia-inducing tumours in mice and humans. *Cancer Res* **55**: 1458–1463
Todorov PT, Cariuk P, McDevitt T, Coles B, Fearon K and Tisdale M (1996) Characterisation of a cancer cachectic factor. *Nature* **379**: 739–742

### Induction of cachexia in mice – reply

**Sir,**

It is certainly correct that the kidney plays an important role in maintaining the concentration of proteins in the extracellular fluid. We have utilized urine as the starting point for the purification for the cachectic factor of $M_r$ 24 000 (Cariuk et al, 1997) because the kidney is so good at filtering out extraneous proteins, so that the purification process is greatly simplified. The reason for not using serum for the routine investigation of the cachectic factor is the low abundance compared with other proteins present. Even in cachexia-inducing tumours the factor represents just 40 ppb of the total protein present (Todorov et al, 1996). This means that serum requires extensive pre-purification of the factor before it can be detected by Western blotting. However, we have no evidence that either serum, or indeed solid tumours, inducing cachexia contain higher molecular weights than the $M_r$ 24 000 form found in urine. The cachectic factor is a highly glycosylated sulphated glucoprotein (Todorov et al, 1997), which is resistant to digestion by pronase, trypsin, chymotrypsin or pepsin (Todorov et al, 1996). This may explain its apparent stability in the body. Although monoclonal antibodies to the factor attenuate the development of cachexia in experimental systems, the road ahead for the treatment of cachexia probably lies with low molecular weight inhibitors of the factor. One such inhibitor, eicosapentaenoic acid (EPA: Lorite et al, 1997), has recently been shown to stabilize weight loss in patients with unresectable pancreatic carcinoma (Wigmore et al, 1996).

*M Tisdale, Pharmaceutical and Biological Sciences, Aston University, Aston Triangle, Birmingham B4 7ET, UK*

**REFERENCES**

Cariuk P, Lorite MJ, Todorov PT, Field WN, Wigmore SJ and Tisdale MJ (1997) Induction of cachexia in mice by a product isolated from the urine of cachectic cancer patients. *Br J Cancer* **76**: 606–613

Lorite MJ, Cariuk P and Tisdale MJ (1997) Induction of muscle protein degradation by a tumour factor. *Br J Cancer* **76**: 1035–1040

Todorov P, Cariuk P, McDevitt T, Coles B, Fearon K and Tisdale M (1996) Characterization of a cancer cachectic factor. *Nature* **379**: 739–742

Todorov PT, Deacon M and Tisdale MJ (1997) Structural analysis of a tumor-produced sulfated glycoprotein capable of initiating muscle protein degradation. *J Biol Chem* **272**: 12279–12288

Wigmore SJ, Ross JA, Falconer JS, Plester CE, Tisdale MJ, Carter DC and Fearon KCH (1996) The effect of polyunsaturated fatty acids on the progress of cachexia in patients with pancreatic cancer. *Nutrition* **12** (suppl): S27–S30

### The effect of three week tamoxifen treatment on oestrogen receptor levels in primary breast tumours: a flow cytometric study

**Sir,**

I was surprised to find an inconsistency in the data presented recently by Brotherick et al 1998). In Figure 1C and D, the authors show gated flow-cytometry histograms supposedly obtained from gates applied to the data in Figure 1A. Close inspection of the data on the *x*-axis, though, clearly indicates that Figure 1C and D were not obtained from the data in Figure 1A. The peak of cells stained with anti-oestrogen receptor antibody (anti-ER) for the cytokeratin-positive cells shown in Figure 1A would occur at approximately channel $1 \times 10^1$, whereas the alleged corresponding peak in Figure 1C occurs at approximately channel $8 \times 10^1$. The same situation applies to the cytokeratin-negative cells shown in Figure 1D, whose peak is at channel $1 \times 10^1$, even though it is clear that the peak of anti-ER-stained cells in Figure 1A occurs far below channel $1 \times 10^1$.

One explanation for this discrepancy is that different samples may have been used in the preparation of this figure, instead of one specific sample (as indicated in both the text and the legend).

Given this flaw, there is no convincing reason to trust the data derived in Figure 2. Altogether, there are insufficient data to support the authors' concluding statement.

*M Sheard*
*Masaryk Memorial Cancer Institute, Zluty Kopec 7, 656 53 Brno, The Czech Republic*

**REFERENCE**

Brotherick et al (1998) *BJC* **77**(10): 1657–1660

### Involvement of radon levels in lung cancer

**Sir,**

Devon, Cornwall and the Isles of Scilly have had low standard mortality rates (SMRs) for lung cancer for a number of years:

| 1994–1996 SMR (95% CI) | Cornwall and Isles of Scilly | South and West Devon | North and East Devon |
|---|---|---|---|
| Male | 92 (85–99) | 86 (80–92) | 74 (68–81) |
| Female | 79 (70–89) | 90 (81–99) | 64 (56–73) |

These low SMRs persist despite a smoking prevalence in the peninsula that is no more than the national average (Torbay and Plymouth Lifestyle Health Survey, 1993) and we have always been doubtful about any major effect of radon on these rates. We had looked forward to the results of the study by Darby et al (1998) as likely to give a definitive answer to this matter, but were disadvantaged by having to comment publicly on a press release by the authors in advance of the paper's publication. Now that we have read the paper we are concerned that the conclusions reached are not justified by the data published in the article. In particular, since the authors state in their conclusion: 'although the confidence intervals for these estimates just include zero, … the combined evidence therefore suggests that a zero effect would not be an appropriate interpretation of the study's results'.

We are concerned that the public may be alarmed unnecessarily, as it seems inappropriate to extrapolate the findings of other studies done in very different circumstances to this particular study. If, as seems likely, the authors are anticipating the outcome of a meta-analysis of all these studies, which may demonstrate a more definite link, then we would welcome this further research, but it does not alter the findings from this study.

It would be helpful if the authors could comment on the points giving us particular concern. Not surprisingly, most of them relate to the ways in which the authors have sought to manage the effect of smoking:

1. The exclusion of smoking-related diseases from the control group leads to an under-representation of smokers in the

© Cancer Research Campaign 1999

control group (Table 3 in Darby et al, 1998). Controls should have been selected to represent those people who would have been represented as cases had they developed the disease and not to represent the entire non-diseased population (Rothman, 1986).

2. Tobacco consumption is estimated from reported smoking habits only, which are known to under-report true smoking status (Hill and Roberts, 1998). There is therefore a potential mis-classification bias with the under-reporting of smoking status greater in cases than controls, which is likely to lead to an underestimation of the effect of smoking in cases.
3. There has been no attempt to measure passive smoking; since the effect of radon is likely to be of the same order of magnitude as that of passive smoking, analysis of its effect would appear essential in order to identify any additional effect of radon.
4. While adjustment for smoking was made in seven categories, only four appear in Table 11 (Darby et al, 1998), which suggests that current smokers apparently have lower excess relative risk than ex-smokers. This would appear to support previous work indicating that many people stop smoking as they become ill. Including recent ex-smokers with people who stopped smoking more then 2–3 years ago would thus appear inappropriate.
5. The authors have not demonstrated 'little confounding between radon and smoking status' as stated in their discussion. It is not clear that stratification is adequate when there has been selection of controls according to the factor in question. The question of effect modification between smoking and radon is of great interest and not adequately answered by including interaction terms within the model (Hill and Roberts, 1998). Residual confounding is particularly important if the exposure has a weak effect.
6. The public health implications of this study are difficult to interpret, as only a small proportion of cases and controls (< 5% of each group) were exposed to levels of radon above the threshold at which the NRPB currently recommends remedial action (200 Bq $m^{-3}$) and an even smaller proportion of lung cancer cases (0.4%) were non-smokers.

Thus, in summary, we believe that differences between the study and control groups, if real, could be explained by the inadequate measurement of tobacco-related behaviour in all the group. As Directors of the local Public Health Departments who have to deal with regular enquiries about radon, it will be difficult to give any weight to the authors' conclusions until we have further comments.

*D Miles[1], J O'Brien[2] and M Owen[3],*
*[1]Department of Public Health Medicine, Cornwall and Isles of Scilly Health Authority; [2]Department of Public Health Medicine, South and West Devon Health Authority; [3]Department of Public Health Medicine, North and East Devon Health Authority, UK*

### REFERENCES

Darby S, Whitley E, Silcocks P, Thakrar B, Green M, Lomas P, Miles J, Reeves G, Fearn T and Doll R (1998) Risk of lung cancer associated with residential radon exposure in south west England: a case control study. *Br J Cancer* **78**: 394–408
Hill A and Roberts J (1998) Body mass index: a comparison between self reported and measured height and weight. *J Public Health Med* **20**: 206–210
Rothman JK (1986) Modern Epidemiology. Little Brown, Boston
Plymouth and Torbay Health Authority (1993) Lifestyle and Health Survey

### Involvement of radon levels in lung cancer – reply

**Sir,**

We can sympathize with Drs Miles, O'Brien and Owen in their puzzlement about the co-existence of relatively low standardized mortality rates for lung cancer in Devon and Cornwall with the high average levels of residential radon. This, however, is neither a new phenomenon, nor even one limited to this country. A similar negative association has been observed in the USA, leading some to think that small doses of ionizing radiation might even be good for you (Lubin, 1998). Interpretation of ecological data on the effects of exposure to residential radon is complicated by the fact that behavioural factors, particularly smoking, but also to some minor extent diet, are so much more important a cause of lung cancer. It is difficult to take these factors fully into account because smoking has such a major effect, increasing the risk by 20-fold or more when cigarettes have been smoked consistently for a long time, and the available data on the prevalence of smoking by amount, type, sex and age are quite inadequate for the purpose. Hence, it has been essential to make direct observations on the effects of residential radon based on studies of individuals and not just to rely on ecological correlations, or on extrapolations made from the experience of miners who have been exposed to similar, or somewhat greater, concentrations in very different circumstances.

In interpreting the data from our own study, it would be bad science to ignore all the other available information. In fact, it would be making the same mistake as the tobacco industry made when its representative claimed that passive smoking must be harmless because one recent European study gave a lower 95% confidence limit to the findings that just included a zero effect. There have now been ten case-control studies of the relationship between radon levels measured in homes and the risk of lung cancer, and in combination they give results very similar to our best estimate and to estimates from the experience of miners, as we noted in our paper, and as shown below:

| | Increase in annual risk of lung cancer per 100 Bq $m^{-3}$ (%) |
|---|---|
| Estimates from miners | +9 |
| Meta-analysis of eight residential studies | +9 |
| Other residential studies | |
| W Germany | +13 |
| SW England | +8 |

 *© Cancer Research Campaign 1999*

All these estimates should probably be increased by about 50%, as shown in our paper, because of uncertainties in the measurement of radon concentrations. As to the specific points that Drs Miles, O'Brien and Owen make about our study, our response is:

1. Our controls were precisely those that 'represent those people who would have been represented as cases had they developed the disease' as half the group have been drawn at random from the relevant population (matched for sex and age) and the other half, the hospital controls (chosen to ensure that they had the same smoking habits as the general population), had almost identical smoking habits. The same result would have been obtained if only general population controls had been used, though with wider confidence limits, because of the smaller amount of data.
2. There is no reason to think that the cases under-reported their smoking habits more than the controls, because our findings for the relative risk of lung cancer by smoking habit were similar to, if not greater than, those recorded in unbiased cohort studies.
3. Exposure to passive smoking was measured and is being reported in a collaborative European study; it had no effect on the results.
4. Table 11 in our original paper (Darby et al, 1998) shows the increased relative risk with radon by smoking category. There is no significant heterogeneity in the findings and they should not be taken to imply that the risk is less for ex-smokers than for current smokers. The much more extensive miners' data suggest that the increased relative risk is greater for non-smokers than for smokers. While only four categories of smokers are shown in the Table, our other three categories (which relate to the amount smoked among current smokers and the time since quitting among ex-smokers) are adjusted for within current and ex-smokers as appropriate. The findings in Table 11 have *no* bearing on the relative risk attributable to smoking and do not support the idea that people stop smoking when they become ill.
5. As mentioned in our paper, the crude increase in risk per 100 Bq $m^{-3}$ is 0.05; this changes by only 0.03 to 0.08 after adjustment for age, sex, county of residence and social class as well as smoking. This demonstrates that there was little confounding between radon and smoking status. Furthermore, seven categories were used in the adjustment for smoking status, and this level of stratification has been demonstrated on the British doctors' data to leave little residual confounding (Breslow and Day, 1980).
6. The public health implications of our paper are quite clear:
   a. it is much more important for smokers to stop smoking than to do anything about the radon concentration in their home
   b. regardless of smoking status, it is worth encouraging people to have the radon concentration in their home measured if they live in an area where the level is likely to be above 200 Bq $m^{-3}$ (the advised action level) and to take action to reduce any high level if it is considered that an increase in the risk of lung cancer of more than about 20–30% is a matter of public health concern
   c. building regulations should be such as to ensure that radon does not seep from underground into newly built houses (which is not difficult to achieve), at least in areas where the concentrations may be high.

Lastly, we note that Drs Miles, O'Brien and Owen would welcome a meta-analysis of all available studies; this is in process. Funding has been obtained from the European Union to undertake a collaborative re-analysis of all 13 European studies currently completed or underway and is being organized by our group in Oxford. A similar collaborative re-analysis (superior to a meta-analysis of published results) is being undertaken in North America, after which it is hoped to merge the two sets of results. It will, however, be some time before the results are available.

*R Doll[1], S Darby[2], E Whitley[2,3], P Silcocks[2,4], B Thakrar[2,5], M Green[6], P Lomas[6], J Miles[6], G Reeves[2] and T Fearn[7]*
*[1]Clinical Trial Service Unit, University of Oxford, Harkness Building, Radcliffe Infirmary, Oxford OX2 6HE, UK; [2]ICRF Cancer Epidemiology Unit, University of Oxford, Gibson Building, Radcliffe Infirmary, Oxford OX2 6HE, UK; [3]Department of Social Medicine, University of Bristol, Canynge Hall, Whiteladies Road, Bristol BS8 2PR, UK; [4]Trent Institute for Health Services Research, Queens Medical Centre, Nottingham NG7 2UH, UK; [5]Glaxo Wellcome, Medical Data Sciences, Greenford Road, Greenford, Middlesex UB6 0HE, UK; [6]National Radiological Protection Board, Chilton, Didcot, Oxon OX11 0RQ, UK; [7]Department of Statistical Science, University College London, Gower Street, London WC1E 6BT, UK*

### REFERENCES

Breslow NE and Day NE (1980) Statistical Methods in Cancer Research Vol 1: The Analysis of Case-control Studies. International Agency for Research on Cancer: Lyon

Lubin JH (1998) On the discrepancy between epidemiological studies in individuals of lung cancer and residential radon and Cohen's ecologic regression. *Health Physics* **75**: 4–10

Darby S, (1998) *Br J Cancer* **78**: 394–408

### Measurement of extravascular drug diffusion in multicellular layers

**Sir,**

A recent study by Phillips et al (1998) describes a simple tissue culture assay for investigating the extravascular penetration properties of anticancer drugs. The reported assay is a modification of the method developed by ourselves (Cowan et al, 1996; Hicks et al, 1997) and Minchinton et al (1997) in which tumour cells are grown as multicellular layers (MCL) on commercially-available Teflon microporous membranes. The ability of drugs to penetrate a MCL separating two compartments can be investigated by adding the compound of interest to the 'donor' compartment and measuring its concentration in the 'receiver' compartment as a function of time.

© Cancer Research Campaign 1999

The approach described by Phillips et al is similar to that used to investigate drug penetration of Caco-2 monolayers, which model the intestinal epithelial barrier (Adson et al, 1994). This method differs from our own in several respects, both experimental and analytical; some of these differences have important implications if the intention is to understand transport in the extravascular compartment of tumours, and thus warrant comment. Phillips et al employ a Transwell vessel suspended in the well of a 24-well plate to investigate penetration. An unstirred donor compartment of 100 μl is used above the MCL, and this vessel is transferred at intervals to a new well containing stirred medium (600 μl) as the receiver compartment. In contrast, our initial studies (Cowan et al, 1996; Hicks et al, 1997, 1998) used an unstirred donor compartment of 500 μl (containing agar to prevent convective mixing) and a stirred receiver compartment of at least 5 ml. As Phillips et al point out, the smaller volumes in their system require less compound, which we acknowledge as a distinct advantage.

The more important experimental difference is that in the Phillips system the donor compartment, although not deliberately stirred, does not contain a gelling agent such as agar to prevent convective mixing. Phillips et al note that addition of agar 'significantly reduces the rate of drug penetration' in the absence of cells. The effect of agar is almost certainly through its prevention of mixing in the donor compartment, which will greatly facilitate mass transfer. It would be impossible to avoid this artifact when the vessel is moved between wells at frequent intervals, as in the Phillips study. In fact the contribution of mixing to intercompartmental transfer is evident in the data presented. We have used numerical methods (Hicks et al, 1997) to model the flux of tirapazamine due to diffusion in the absence of cells under the conditions used by Phillips et al, using the diffusion coefficient of tirapazamine in culture medium at 37°C ($8.75 \times 10^{-6}$ cm$^2$ s$^{-1}$; Hicks et al, 1998). We assume that the microporous Teflon membrane supporting the MCL is only 10 μm thick, with 100% porosity, and that there are no unstirred boundary layers. Even with these unrealistically favourable assumptions, the half-time for purely diffusive transfer between an unstirred donor compartment of 100 μl and a stirred receiving compartment of 600 μl (in the absence of an MCL) would be 30 min, whereas Figure 4 of Phillips et al (1998) shows a half-time of approximately 5 min. Using these same parameters but assuming efficient mixing in the donor compartment gives slightly faster transfer (half-time 3.5 min) than observed. It is thus probable that convective mixing in the donor compartment in the Phillips assay is intermediate between the no-mixing and fully mixed extremes. This gives a situation that is impossible to model with precision.

But does the difficulty in extracting the underlying diffusion parameters by modelling really matter? It could be argued that, even if the extent of mixing is ill-defined, providing it is reasonably reproducible an appropriate rank ordering of compounds could still be achieved. This brings us to a consideration of the analytical approach taken by Phillips et al, which uses a compartmental model and characterizes mass transfer between the two compartments as a first-order process. The parameter determined is the half-time or first-order rate constant for transfer, which would appear to provide a simple means for ranking compounds. This approach is appropriate in the Caco-2 model, where the objective is simply to quantitate mass transfer between compartments. However, in the MCL model the objective is to infer, from the net flux, the concentration gradient *within* the intervening tissue compartment. This requires modelling the flux explicitly as diffusion to extract the underlying transport parameters (diffusion coefficient $D$ and reaction rate constants), allowing use of the same distributed parameter model to calculate pharmacokinetic parameters within the extravascular compartment of a tumour. The quite different boundary and initial conditions in vivo make it very

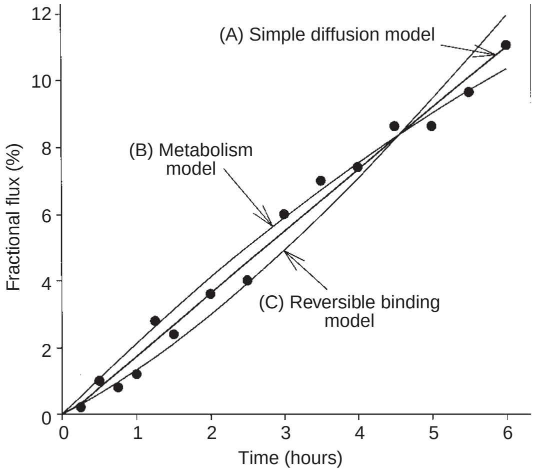

**Figure 1** Hypothetical flux data (●) for a MCL (200 μm thick) with the donor and receiver compartments (5 ml each) both well-stirred. The ordinate is the concentration in the receiver as a percentage of that expected at equilibrium (ignoring any loss due to reaction). Models are fitted, assuming a homogeneous MCL, to a generalized reaction-diffusion equation:

$$\frac{\partial c}{\partial t} = D\frac{\partial^2 c}{\partial x^2} - k_{met}c - k_1 c + k_{-1}\, c_b$$

where $D$ is the diffusion coefficient for free drug, $k_{met}$ is the first order rate constant for metabolism, $k_1$ and $k_{-1}$ are the forward and backward rate constants for reversible binding, and $c$ and $c_b$ are the concentrations of free and bound drug respectively. The fitted parameters for the three models are shown in Table 1

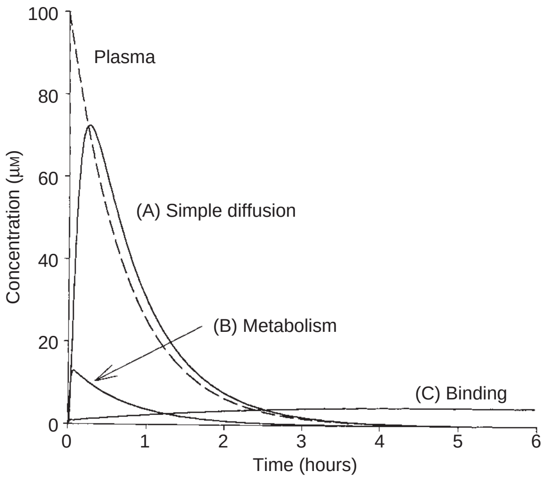

**Figure 2** Simulated concentration-time curves in a tumour microregion 150 μm from blood vessels (——) for a drug that is cleared from plasma with first-order kinetics (initial concentration 100 μM, half-life 30 min; – – –), using the transport parameters for the three models fitted to MCL flux data in Figure 1. The geometry is approximated as diffusion into a plane slab from both sides, which is a compromise between radial inward and radial outward diffusion. The calculated pharmacokinetic parameters for the central plane are summarized in Table 1

 *© Cancer Research Campaign 1999*

**Table 1** Fitted parameters for modelling of MCL flux (see Figure 1), and derived pharmacokinetic parameters for free drug at the centre of an extravascular region in a tumour (see Figure 2)

| Model | Parameter values in the MCL used to model flux | | | Pharmacokinetic parameters in extravascular compartment | | |
|---|---|---|---|---|---|---|
| | $D$ ($cm^2\,s^{-1}$) | $k_{met}$ ($min^{-1}$) | $k_1/k_{-1}$ | $C_{max}$[a] (μM) | Time to $C_{max}$ (min) | $AUC_t/AUC_p$[b] |
| (A) Simple diffusion | $2.5 \times 10^{-7}$ | 0 | 0 | 72.5 | 15 | 1.00 |
| (B) Diffusion with first order metabolism | $5 \times 10^{-7}$ | 0.95 | 0 | 12.8 | 4 | 0.14 |
| (C) Diffusion with reversible binding | $5 \times 10^{-7}$ | 0 | 200 | 4.3 | 288 | 0.99 |

[a]Maximum concentration. [b]AUC (area under the concentration-time curve) at the centre of the tumour microregion as a fraction of the plasma AUC.

difficult to use in vitro compartmental parameters to predict pharmacokinetics within tissue, particularly since the experimental half-life will be a function of thickness of the MCL (as noted by Phillips et al) and will also depend on other details of the experimental model. In contrast, diffusion parameters, if properly fitted, are independent of these factors and can be applied directly to describe the pharmacokinetics at any required position in the extravascular compartment.

The importance of this becomes clearer with an example. Figure 1 shows hypothetical (but typical) MCL flux data for a drug in culture medium using the diffusion chamber apparatus developed in Minchinton's laboratory (Kyle and Minchinton, 1998). In our example the drug penetrates the MCL slowly, with a half-time for intercompartmental transfer of about 24 h, but this value does little to tell us whether the drug penetrates tumour tissue adequately. If the flux is modelled explicitly as Fickian diffusion, then the slow flux can be considered to result from (a) a low $D$ in the MCL, (b) irreversible reaction in the MCL (i.e. drug metabolism) or (c) reversible reaction, such as non-covalent binding to immobile macromolecules (e.g. binding of intercalators to DNA). There are enough adjustable parameters in the binding models (Table 1) that all three diffusion models fit the data equally well (similar sum of squares) in this case. However, the three models have very different implications for extravascular diffusion, as illustrated in Figure 2. This shows the concentration-time profile for free drug in the centre of a 300 μm-thick tumour region (simplified, planar geometry is used in this example, but a qualitatively similar picture is obtained with more realistic geometries), assuming a plasma half-life of 30 min. The pharmacokinetics in this microregion differ dramatically depending whether the impediment to penetration in the MCL comes from a genuinely low $D$, a higher $D$ with metabolism (reduced AUC) or a higher $D$ with reversible binding (lowered $C_{max}$). The pharmacodynamic implications will also, of course, be very different for the three situations, and will depend (amongst other things) on whether there are threshold effects for the desired biological responses. Measurement of flux across MCL cannot easily distinguish these three extravascular transport models, although metabolism results in a concave downwards flux curve, while for reversible binding the curve is concave upwards because flux speeds up as binding sites become filled. In some cases this curvature is sufficient to make it obvious that binding or metabolism is at work, as we illustrate in Hicks et al (1997, 1998) respectively. However, in general it will be more reliable to constrain the choice of models using independent information about the metabolic and cell uptake/binding characteristics of the drug in question.

In the Phillips study the demonstration that transfer of EO9 has a longer half-time for intercompartmental transfer than TPZ (possibly limited by metabolism), but shorter than doxorubicin (probably limited by reversible binding), cannot be used to draw firm conclusions about the adequacy of its extravascular penetration properties. The observation that dicoumarol increases flux by about 30% does suggest that penetration could be partially limited by drug metabolism, and this might well compromise delivery to cells distant from vessels. But an explicit reaction–diffusion model is needed to enable predictions under typical in vivo conditions. We fully concur with Phillips et al that drug penetration is an important issue in cancer chemotherapy, and that experimental models such as MCL can contribute to understanding this problem, but it may be premature to conclude that poor penetration of EO9 is responsible for its lack of activity in clinical trials. Such an interpretation is not easy to reconcile with the anti-tumour activity of EO9 in mice, including against human tumour xenografts (Roed et al, 1989; Hendriks et al, 1993) and hypoxic cells in the KHT tumour (Adams et al, 1992), despite a plasma half-life threefold less in mice than humans.

The experimental model developed by Phillips et al has some very attractive features in its miniaturization and convenience, and could be adapted to overcome the above concerns by using either agar in the donor compartment, or deliberate and efficient mixing (as is used with the Caco-2 model). Any intermediate condition will be difficult to reproduce, and is not amenable to modelling. Extraction of the underlying transport parameters by modelling the flux data is of fundamental importance in developing an understanding of the pharmacokinetics of anticancer agents in the extravascular compartment of tumours.

*WR Wilson and KO Hicks*
*Auckland Cancer Society Research Centre, The University of Auckland, Private Bag 92019, Auckland, New Zealand*

**REFERENCES**

Adams GE, Stratford IJ, Edwards HS, Bremner JCM and Cole S (1992) Bioreductive drugs as post-radiation sensitizers: comparison of dual function agents with SR 4233 and the mitomycin C analogue EO9. *Int J Radiat Oncol Biol Phys* **22**: 717–720

Adson A, Raub TJ, Burton PS, Barsuhn CL, Hilgers AR, Audus KL and Ho NFH (1994) Quantitative approaches to dilineate paracellular diffusion in cultured epithelial monolayers. *J Pharm Sci* **83**: 1529–1536

Cowan DSM, Hicks KO and Wilson WR (1996) Multicellular membranes as an in vitro model for extravascular diffusion in tumours. *Br J Cancer* **74** (suppl. 27): S28–S31

Hendriks HR, Piazo PE, Derger DP, Kooistra KL, Bibby MC, Boven E, Dreef-van der Meulen HC, Henrar REC, Fiebig HH, Double JA, Hornstra HW, Pinedo HM, Workman P and Scartsmann G (1993) EO9: a novel bioreductive alkylating indoloquinone with preferential solid tumour activity and lack of bone marrow toxicity in preclinical models. *Eur J Cancer* **29A**: 897–906

Hicks KO, Ohms SJ, van Zijl PL, Denny WA, Hunter PJ and Wilson WR (1997) An experimental and mathematical model for the extravascular transport of a DNA intercalator in tumours. *Br J Cancer* **76**: 894–903

Hicks KO, Fleming Y, Siim BG, Koch CJ and Wilson WR (1998) Extravascular diffusion of tirapazamine**:** effect of metabolic consumption assessed using the multicellular layer model. *Int J Radiat Oncol Biol Phys* (in press)

Kyle AH and Minchinton AI (1999) Measurement of delivery and metabolism of tirapazamine to tumour tissue using the multilayered cell culture model. *Cancer Chemother Pharmacol* **43**: 213–220

Minchinton AI, Wendt KR, Clow KA and Freyer KH (1997) Multilayers of cells growing on a permeable support: an in vitro tumour model. *Acta Oncol* **36**: 13–16

Phillips RM, Loadman PM and Cronin BP (1998) Evaluation of a novel in vitro assay for assessing drug penetration into avascular regions of tumours. *Br J Cancer* **77**: 2112–2119

Roed H, Aabo K, Vindelov L, Spang-Thomsen M, Christensen IJ and Hansen H (1989) In vitro and in vivo evaluation of the indoloquinone EO-9 (NSC 382 459) against human small cell carcinoma of the lung. *Eur J Cancer Clin Oncol* **25**: 1197–1201

### Seasonal variations in the presentation and growth of thyroid cancer

**Sir,**

We read the article by Akslen and Sothern (1998) with interest; in particular, the section concerning the presentation at clinic of the cases with thyroid cancer where the authors note: '… significantly more cases presenting during the late autumn and winter'. Without disputing this finding, we would nevertheless like to point out how this conclusion might be refined.

We first note that in a non-leap year and in the absence of any seasonal effect, one would expect almost 11% more cases in January, which has 31 days, than February, which has 28 days. Thus the calendar month data given in Akslen and Sothern (1998, Figure 1B) and reconstructed in Table 1 below, should be adjusted to 'months' of equal duration which can be taken for convenience as each of 30 days. This adjustment is carried out in two stages. First, the number of observations in 31-day calendar months, such as in each of the 16 January's of the study period, are reduced by (30/31), February increased by (30/28) in the 12 non-leap years and by (30/29) in the 4 leap years.

The 30-day months, such as April, remain unadjusted. The corresponding adjusted figures are given in column 3 of Table 1. However, the frequencies in each 30-day month no longer sum to the original 2627 patients. The second stage multiplies the frequencies of column 3 by 2627/2595.98 = 1.01195 to account for this. The adjustment clearly gives a greater emphasis to February by increasing the count from 232 to 249.44 while decreasing those of January and March.

These data can be illustrated graphically in a rose diagram format (Figure 1) where each petal is of a 'standard' month or 360/12 = 30°. In this diagram, the square root of $f$ is utilized to preserve equal areas for each unit of frequency, as is the case for a conventional histogram.

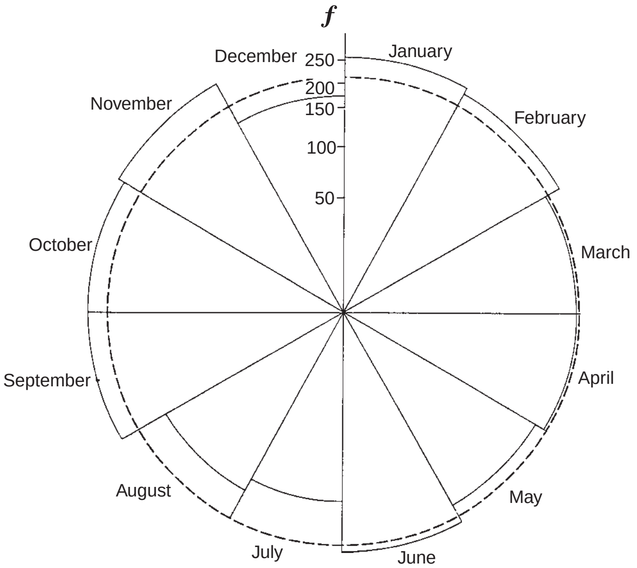

**Figure 1** Rose diagram of the monthly distribution of thyroid cancer. (The hatched circle denotes the expected value per month if seasonality is absent)

**Table 1** Monthly presentation of thyroid cancers (after Akslen and Sothern, 1998, Figures 1A and B)

| Month | Number of Patients (*n*)[a] | First adjustment | Final adjusted frequency (*f*) |
|---|---|---|---|
| January | 272 | 262.77 | 265.91 |
| February | 232 | 246.49 | 249.44 |
| March | 217 | 210.12 | 212.63 |
| April | 210 | 210.17 | 212.68 |
| May | 200 | 193.69 | 196.00 |
| June | 238 | 237.67 | 240.51 |
| July | 115 | 111.54 | 112.88 |
| August | 152 | 146.80 | 148.56 |
| September | 261 | 260.70 | 263.82 |
| October | 269 | 259.88 | 262.99 |
| November | 279 | 278.87 | 282.20 |
| December | 183 | 177.26 | 179.38 |
| Total | 2627 | 2595.98 | 2627.00 |

[a]Estimated from Figure 1 of Akslen and Sothern (1998).

*© Cancer Research Campaign 1999*

Further improvement in the analysis can be made by utilizing the actual dates of presentation of the patients with the thyroid cancer, rather than merely using the corresponding month. This more precise analysis, described by Machin and Chong (1998) following the earlier suggestions of Mardia (1972), enables the estimation of the peak date of presentation. If this approach is applied (see Machin and Chong for details) to the grouped data of Table 1, then the estimate of the peak date is 22 December (week 51). However, quoting the 'day' of peak here when using months rather then the day as the unit for analysis may imply rather spurious accuracy. The analysis based on individual dates also allows estimation of a confidence interval (CI) for the peak date. However, the method of calculation used for the CI depends both on the sample size and the magnitude of the corresponding peak (Fisher, 1993) and requires the individual dates of presentation. Utilizing the grouped data and a bootstrap approach we obtained a 95% CI from 3 December to 10 January, but this is not likely to be very reliable.

One rather unfortunate consequence of using monthly data is then to test for the presence of seasonality by calculating a $\chi^2$ test with 11 degrees of freedom (*df*). However, Newcombe (1983) pointed out that this test only tests for departures from a uniform distribution of cases throughout the year and not specifically for the presence of a single peak. The test effectively compares each frequency (*n* of Table 1) with its expected value; calculated under the null hypothesis of a uniform distribution of cases throughout the year. The authors obtained $\chi^2 = 119.3$ with $P < 0.00001$ which, although clearly statistically significant, does not imply that there is therefore a (single) peak (date) of presentation.

A more appropriate test for the presence of a single peak has been described by Mardia (1972; see Machin and Chong); this utilizes the individual dates but can be calculated for grouped data following the adjustments to the frequencies we have described in Table 1. This $\chi^2$ test has only 2 *df* and tests specifically for the presence of a single peak in the data. For the data given here, $\chi^2_{\mathrm{Mardia}} = 38.01$, $df = 2$ and $P < 0.00001$, and this provides clear evidence of a (single) peak.

The definition of the (northern hemisphere) seasons used by Akslen and Sothern (1998) are the four quarters of the year, thus Winter is January, February and March. These 'seasons' differ from those defined by, for example, Bounameaux et al (1996) where Winter begins in December. This difference in season definition is one further difficulty that arises if data are grouped for analysis and presentation. Such coarse groupings may make comparisons between studies in different geographical locations less meaningful.

In summary, although our assessment of these data has not materially affected the conclusions of the authors, they do indicate more precisely that the peak occurs in the December to January period and the test we describe is specifically for that purpose. We would reiterate Gilman et al (1998) and stress the importance of the optimal use of the statistical techniques available if we are to successfully identify aetiological factors that may be relevant to the understanding of the disease in question.

*D Machin[1] and SF Chong[1,2]*
*[1]National Medical Research Council, Clinical Trials & Epidemiology Research Unit, 10 College Road, Singapore 169851, [2]Department of Clinical Research, Ministry of Health, Singapore General Hospital, Singapore 169608*

#### REFERENCES

Akslen LA and Sothern RB (1998) Seasonal variations in the presentation and growth of thyroid cancer. *Br J Cancer* **77**: 1174–1179

Bounameaux H, Hicklin L, Desmarais S (1996) Seasonal variation in deep vein thrombosis. *Br Med J* **312**: 284–285

Fisher NI (1993) *Statistics of Circular Data*. Cambridge University Press: Cambridge

Gilman EA, Sorahan T, Lancashire RJ, Lawrence GM and Chong KK (1998) Seasonality in the presentation of acute lymphoid leukaemia. *Br J Cancer* **77**: 677–678

Machin D and Chong SF (1998) On the detection of the seasonal onset of disease. *J Epidemiol Biostat* (in press)

Mardia KV (1972) *Statistics of Directional Data*. Academic Press: London

Newcombe RG (1983) Cold weather and testicular torsion. *Br Med J* **287**: 359

### Seasonal variations in the presentation and growth of thyroid cancer – reply

**Sir,**

With regards to comments by Machin and Chong on our assignment of number of cases of thyroid cancer to calendar month, rather than to 12 equal intervals of the year (Akslen and Sothern, 1998), we are fully in concurrence that the latter would allow for a more accurate comparison of seasonal patterns, since grouping data solely by month may allow for slightly larger totals in months with 31 days and slightly fewer in days with 30 days, or in February with only 28 or 29 days. However, only month of diagnosis for the 2627 cases of thyroid cancer was entered from the Norwegian Cancer Registry. The point of Machin and Chong is well taken that careful records should be used in such epidemiologic studies in order to accurately detect and describe seasonal and other predictable variations in disease, as shown in a recent study on peptic ulcer (Svanes et al, 1998), where the results were reported as incidence per month using 12 equal portions of the year. In the latter study, we were also able to look at day of the week and even clock time.

For analyses of the thyroid cancer incidence data, we assigned monthly totals to the middle of each month. With the data so coded, it was impossible to reassign some of the cases to another month, except by such a method proposed by Machin and Chong which adjusts the monthly totals proportionately. Regarding the grouping of data by season, the choice of 3-month totals beginning with Jan–Feb–Mar was somewhat arbitrary, since we were not able to get actual 'seasonal' totals by beginning the first interval at the

© Cancer Research Campaign 1999

Winter solstice (22 December); our data were assigned to the 15th of each month (14th for February). We did group incidence data in 3-month segments beginning with Dec–Jan–Feb and actually got a better chi-square result ($\chi^2 = 72.1$) than we reported with intervals beginning with Jan–Feb–Mar ($\chi^2 = 35.6$), but the *P*-values were < 0.0001 for each of these analyses. The use of the 3-month groupings beginning with Jan–Feb–Mar made sense for the area of the world where the data were collected, which is subjected to large changes in daylight throughout the year. During the seasons we chose, the daylight is returning, but there is still more dark than light each day (Jan–Feb–Mar), the daylight goes from half the day to nearly the whole day (Apr–May–Jun), the amount of daylight shortens to half the day (Jul–Aug–Sep), and the amount of daylight decreases to nearly total darkness (Oct–Nov–Dec). It is clear that seasonal patterns may not be easy to compare between hemispheres and between studies using different 3-month spans, and we therefore emphasize the importance of also showing 'monthly' values that are properly computed.

When we analysed our data several years prior to publication, we were not aware of a newer technique to compute a time of peak in incidence along with a confidence interval. It is true that such data should be analysed by the 'optimal … statistical techniques available', even though these authors state that their 'assessment of the data has not materially affected the conclusions of the authors', and we will be interested in learning about the technique of Machin and Chong (1998) when it is published. We are familiar with an earlier statistical procedure that tests for a peak in a time series (Savage et al, 1962), but this is mostly used when a rhythmic pattern is not pronounced and does not provide a confidence interval. In a rhythmic time series, usually the peak of a cosine model with the proper components to describe the waveform will accurately locate the peak in the data (Tong et al, 1977), and this is what we tried to do. However, periods harmonic to 1-year were not significant for the incidence data, and consequently only the 1-year cosine result was used. Of note, both our estimate of the highest incidence and that of Machin and Chong, along with their 95% confidence interval, occurs in December, but inspection of the monthly totals clearly shows a drop in incidence in December and highest values in Sep–Oct–Nov. Therefore, we emphasized that the highest incidence occurred in the late Fall–Winter, rather than near a single date.

Finally, we wish to thank Machin and Chong for asking us for the chance to apply their technique to our data. In so doing, we found a coding error for the Jan monthly incidence total which caused the graph in Figure 1B in our paper (Akslen and Sothern, 1998) to be in error in that the first dot at 274 should have been graphed at 241. While $\chi^2$ analyses and conclusions remain unaltered, the *P*-value for the 1-year cosine should have been given as 0.253, the mesor as 219 ± 12, the amplitude as 32 ± 17 and the acrophase as 15 Dec in Table 1. We regret the error.

*LA Akslen[1] and RB Sothern[2]*
*[1]Department of Pathology, The Gade Institute, University of Bergen, Bergen, Norway; [2]Biorhythmometry, College of Biological Sciences, University of Minnesota, St Paul, MN 55108, USA*

### REFERENCES

Akslen LA and Sothern RB (1998) Seasonal variations in the presentation and growth of thyroid cancer. *Br J Cancer* **77**: 1174–1179

Machin D and Chong SF (1998) On the detection of the seasonal onset of disease. *J Epidemiol Biostat* **3**: 385–394

Savage IR, Rao MM and Halberg F (1962) Test of peak values in physiopathologic time series. *Exp Med Surg* **20**: 309–317

Svanes C, Sothern RB and Sørbye H (1998) Rhythmic patterns in incidence of peptic ulcer perforation over 5.5 decades in Norway. *Chrono Intl* **5**: 241–264

Tong YL, Nelson WL, Sothern RB and Halberg F (1977) Estimation of the orthophase – timing of high values on a non-sinusoidal rhythm – illustrated by the best timing for experimental cancer chronotherapy. In: Proc XII Intl Conf Intl. Soc Chronobiology, Washington, DC, 10–13 Aug, 1975. Il Ponte: Milan: 765–769

*© Cancer Research Campaign 1999*

